# Comparison of phenothrin mousse, phenothrin lotion, and wet-combing for treatment of head louse infestation in the UK: a pragmatic randomised, controlled, assessor blind trial

**DOI:** 10.12688/f1000research.2026.1

**Published:** 2014-07-10

**Authors:** Ian F. Burgess, Christine M. Brown, Pat Nair

**Affiliations:** 1 Medical Entomology Centre, Insect Research & Development Limited, Cambridge, CB25 9AU, UK; 2 Consultant in Communicable Disease Control, Bedfordshire Health Authority and Bedfordshire Family Health Authority, Luton, LU1 3AN, UK

## Abstract

In this investigation of effectiveness of an alternative pediculicide dosage form, we recruited 228 children and 50 adult participants from Bedfordshire, UK, to a randomised, controlled, assessor blind trial comparing two insecticide products with mechanical removal of lice as a control group.  Participants using insecticide were treated with either the investigative 0.5% phenothrin mousse, for 30 minutes, or 0.2% phenothrin lotion, for 2 hours as the reference product.  Both treatments were applied only once, followed by shampoo washing.  Those treated by wet-combing with conditioner were combed 4 times over 12 days.  Parents/carers carried out the treatments to mimic normal consumer use.  The outcome measure was the absence of lice, 14 days after treatment for the insecticides, and up to 14 days after completion of combing.  Intention to treat analysis of the outcomes for 275 participants showed success for phenothrin mousse in 21/105 (20.0%), in 23/107 (21.5%) for phenothrin lotion, and in 12/63 (19.1%) for wet-combing.  People receiving mousse were 1.07 (95% CI, 0.63 to 1.81) times more likely to still have lice after treatment compared with those treated with lotion. The group of participants who received the wet combing treatment were 1.13 (95% CI, 0.61 to 2.11) times more likely to still have lice after the treatment.  None of the treatments was significantly (p < 0.05) more effective than any other. This study was carried out in an area where moderate resistance to phenothrin was demonstrated after the study by using a bioassay.  Analysis of post treatment assessments found that failure of insecticides to kill louse eggs had influenced the outcome.

## Introduction

During the early 1990s, a number of synthetic pyrethroid-based formulations for treating head louse infestation were introduced into the British market. The majority of these used *d*-phenothrin as the active substance in a variety of dosage forms ^1^. In 1997 a phenothrin mousse was developed based on a concept developed in Australia using natural pyrethrum ^2^. The aim of the new product was designed to be more manageable during application and thus gain greater consumer acceptability than existing preparations. Laboratory studies of phenothrin lotion had indicated a high level of activity for the insecticide, so theoretically it could be incorporated into a formulation requiring a shorter application time ^1^. However, at this time there was increasing evidence of insecticide resistance in several areas of the UK ^3, 4^, in parallel with a renewed consumer interest for treating head louse infestation by combing, either as the principal measure or as a component of conventional insecticide treatment. At the time of the study the most widely promoted combing method was “Bug-Busting” (Community Hygiene Concern, London), which used a fine toothed plastic comb for wet combing with conditioner, repeated at 3–4 day intervals for 2 weeks. It suggested the first combing could remove all lice so only newly hatched nymphs would be found during subsequent combing sessions before they could mature and lay eggs ^5^. Before our investigation only two studies of wet-combing had been conducted. In one Bug-Busting was half as effective as two applications of malathion lotion ^6^. A second found it more effective than permethrin creme rinse, but the dropout rate from both treatments made the interpretation of the results difficult ^7^.

We performed a pragmatic, observer blinded, three armed clinical trial analysed by intention to treat, comparing single applications of 0.5% *d*-phenothrin mousse or 0.2% *d*-phenothrin lotion with the Bug-Busting protocol of wet-combing with conditioner. The study was designed to evaluate the effectiveness of each treatment when in use by the public.

## Methods

### Participants

We recruited participants (children and adults) to the study from respondents to an information letter distributed through schools or via general practitioners associated with Bedfordshire Health. Prospective participants or their parents/carers telephoned the study co-ordinator to make an appointment for a home visit by a trained agency nurse. Most visits were within 24 hours unless requested at a different time. Nurses followed a standard approach to check for living lice using a plastic detection comb (Albyn of Stonehaven Ltd, Stonehaven, Scotland). If moving head lice were found, and the individual was 4 years or over, they were invited to join the study. Prospective participants were conducted through a standard consent procedure in which the content of information sheet was explained verbally and the parent/carer confirmed that they understood the function, processes, and commitments of the study before signing the consent form, which was witnessed by an independent adult. Participants were individually assigned a randomised treatment. All other household members were offered examination and, if found to be infested with head lice, were given the opportunity to join the study.

Prior to inclusion in the study, all participants provided baseline data including: age, gender, ethnicity, hair characteristics including length, thickness, degree of curl, and previous pediculicide use. Inclusion criteria required availability for up to 28 days to accommodate each of the possible treatment regimens and a suitable adult available to perform or assist in application of the treatment. Candidates excluded from this study were: pregnant or nursing mothers; anyone who had bleached, colour-treated or permanently waved their hair; used pediculicide, had been treated with antibiotics, or had participated in a clinical trial during the 4 weeks prior to this trial. In addition anyone with sensitivity to any pyrethroid insecticide or chrysanthemums; receiving treatment for asthma; suffering from a persistent skin disorder of the scalp (other than head lice); or had already participated in this study; was also excluded.

Household members with lice who either did not wish to participate or who failed to satisfy the inclusion criteria were given advice about appropriate treatment methods. Any participants found with lice after completion of the study period were supplied with 0.5% malathion lotion (Suleo-M lotion, Seton Healthcare Group plc, Oldham, UK) in conformity with the Bedfordshire Health policy for treatment. No payment was offered for participation.

### Ethics

Ethical approval was granted by both the South Bedfordshire and North Bedfordshire Local Research Ethics Committees of Bedfordshire Health, one of the conditions of which was that the data would be published in the public domain. The study was registered with the Current Controlled Trials database ( ISRCTN73201839). See Supplementary files for the study protocol.

The study was conducted in compliance with Good Clinical Practices, and in conformity with the principles of the Declaration of Helsinki and European Standard, EN540: Clinical investigation of medical devices for human subjects. Written and witnessed informed consent was obtained from the participants and parents or guardians of children under 18 years of age.

### Treatments

The nurses explained how to apply the treatment to the parent or carer and also gave a printed copy of these instructions. The parent then applied the treatment in the presence of the investigator. The nurses were instructed to answer any questions, and to note these, but not to intervene if any error was observed during treatment. This was intended to represent the clinical situation.

We supplied one group of participants with 0.5% *d-*phenothrin mousse in 50ml butane pressurised containers, with a canula fitting to allow direction of the mousse during delivery (Full Marks Mousse, Seton Healthcare Group plc, Oldham, UK). This also contained citrate buffered water, ethanol, and emulsifying wax. We supplied the second group with 0.2% *d-*phenothrin water/isopropanol lotion in 50ml glass bottles with a dropper aperture (Full Marks Lotion, Seton Healthcare Group plc). Carers applied the products to dry hair to saturate the hair and scalp. We made available as many containers of product necessary to comply with the instructions. People using mousse washed it from the hair with shampoo after 30 minutes. Those treated with the lotion left it on the hair for 2 hours before shampooing. We supplied both groups with a non-medicated shampoo (L’Oréal Children’s Shampoo, L’Oréal (UK) Ltd, London, UK) for this. Both groups received a single application of treatment.

We supplied the third group of participants with a “Bug Buster” pack (Community Hygiene Concern, London, UK) for performing the wet-combing technique. We also supplied a bottle of the same non-medicated toiletry shampoo and four 60ml bottles of conditioner rinse (one for each treatment day) (L’Oréal Children’s Shampoo and L’Oreal Conditioner, L’Oréal (UK) Ltd,), and a diary card. Parents/carers were instructed to wash the participant’s hair with the shampoo, massage in a generous amount of conditioner, and comb through the hair systematically from scalp to tip with the louse removal comb provided in the pack. They wiped the comb on a paper towel between strokes. Combing was repeated at regular intervals for 2 weeks (days 0, 4, 8, 12 of the study). We asked carers to fix any lice found during the combing onto the diary card using clear cellulose adhesive tape and to record how much time was spent combing.

### Outcome measures

We made mousse and lotion treatment follow ups on days 4, 7, 10, and 14 and those for wet-combing on days 14, 21, and 28 after commencement of treatment. Day 14 was used as the point for measure of primary outcome for the insecticide groups. However, in the wet-combing group it was possible there could be viable eggs present on day 14 and therefore follow up examinations were also conducted on days 21 and 28 to detect any emerging nymphs or other lice missed at day 14. During each of the follow up assessment examinations, any lice found by detection combing were removed and fixed to the case record using clear cellulose adhesive tape. These were later examined in the laboratory to determine the gender or development stage of each insect.

Nurses collected the containers of mousse and lotion after treatment so that the quantities used could be measured. Bottles of conditioning rinse from the wet combing group were also collected for measurement after the final assessment on day 28, although some of these were mislaid by participants.

### Sample size

We estimated sample sizes to show a difference between wet combing and phenothrin lotion treatment with 95% confidence, 90% power, and equivalence between the two phenothrin groups to within 20%. For this calculation we conservatively estimated that the phenothrin products would exhibit approximately 80% effectiveness and wet combing 50% success. Sample size calculations were made by the sponsor’s consultant statistician who estimated a minimum sample size of 104 participants in each phenothrin treated group and 58 participants treated with wet combing (266 evaluable participants) would satisfy this probability with greater than 90% power.

### Randomisation and blinding

A computer generated list, prepared by the sponsor’s statistician, was used for randomisation of treatments, made up of balanced blocks of 133 treatment allocations with a relative frequency for each of the treatments of 52:52:29. Randomisation was by individual so that different members of a household could receive different treatments. Nurses involved in recruitment were supplied with envelopes in batches of ten and asked to issue them sequentially. Investigators, who were unaware of which treatment had been used, made follow up examinations using plastic detection combs to check for the presence of living lice. On day 4, 7, and 10 these assessors were a different group of agency nurses. On days 14, 21, and 28 the assessing investigators were from the Medical Entomology Centre (IFB and CMB).

### Statistical analysis

We analysed for differences between groups based on the intention to treat (ITT) population and tested equivalence only using the per-protocol (PP) population. We calculated differences in cure rate using a chi-squared test and equivalence to within 20% based on the 95% confidence limits derived from the normal approximation to the binomial distribution. The initial analyses for the sponsor were performed by the consultant statistician (PN Lee Statistics and Computing Ltd, Sutton, UK) using bespoke software. Post hoc analyses performed by the investigators employed Epi-Info version 6, OXSTAT II version 1.11, and purpose built spreadsheet calculators. Differences between groups in baseline characteristics, safety, acceptability, and efficacy were tested using Fisher’s exact test for yes/no variables and the Mann-Whitney U test for ranked variables.

### Tests for resistance

During the course of the study a high level of treatment failure was observed in the insecticide groups. We collected samples of lice at the final assessment from five participants from different parts of the study area for a bioassay evaluation for sensitivity to phenothrin. The insects were placed on treated or control filter papers using a method previously described for tests of permethrin sensitivity ^3^. Each treated filter disc was impregnated with 500 µl of 2% *d*-phenothrin solution, giving an insecticide deposition rate of 157 μg cm ^-2^. The mortality outcomes of the tests were compared, with a baseline sensitivity obtained using laboratory-reared, insecticide sensitive, body/clothing lice by means of log-probit analysis using LDP Line software.

## Results

### Participants

**Figure 1.**
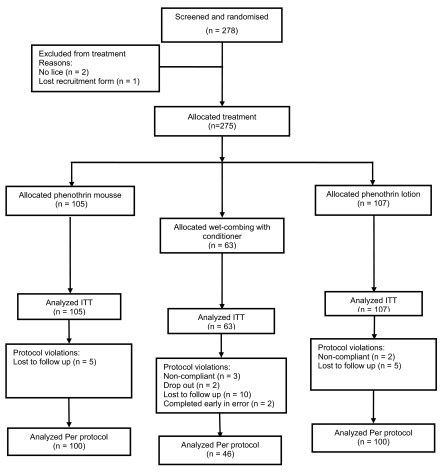
Flowchart of participants through the study.

The study was conducted between June 1997 and March 1998, during which informed consent was obtained for 228 children and 50 adults to participate ( Figure 1). Two people were excluded from further analysis as, upon inspection, no live lice had been found. The recruitment case record form for one other participant was lost so this case was also excluded from the study.

The 275 participants were randomly assigned to one of the treatment groups: 105 received phenothrin mousse, 107 phenothrin lotion, and 63 were allocated to the wet-combing treatment. Of these participants, 246 (89.5%) (100 mousse, 100 lotion, and 46 wet-combing) completed the trial with adequately complete follow-up data sets ( Figure 1 and Dataset 1). From the original study group, 5 participants (2 treated with lotion and 3 wet-combing) were non-compliant and excluded from the per-protocol analyses. Non-compliance involved additional combing or other unauthorised treatments (2 receiving lotion and 2 wet-combing) and one participant on wet-combing shaved his head. There were 24 other withdrawals: 2 people on wet-combing dropped out; 20 were lost to follow-up (5 from the mousse group, 5 from lotion, and 10 from wet-combing); and 2 people from the wet-combing group were not allocated Day 21 or Day 28 appointments in error following a communication failure between the study coordinators and the investigators conducting final assessments. Two of those lost to follow up were due to bereavement and the two drop outs chose not to continue in the study. The rate of protocol violation/withdrawal was significantly higher (p < 0.001) in the wet-combing group than the phenothrin-treated groups.

Of the 275 people known to satisfy the inclusion and exclusion criteria, 62 (23%) were male ( Table 1). The percentage distribution of males was similar for the two phenothrin groups, 18% and 21% respectively, but higher in the wet-comb group (33%). This difference in proportion of males between wet-comb group and mousse-treated group was statistically significant (p < 0.05) and the difference between wet-comb group and the lotion-treated group was nearly significant (0.05 < p < 0.1).

**Table 1.** Demographic characteristics of the intention to treat population.

Characteristic		Mousse	Lotion	Wet-comb	Total
Number of participants		105	107	63	275
Age	4–7	45 (42.9%)	42 (39.3%)	27 (42.9%)	114 (41.5%)
	8–12	36 (34.3%)	40 (37.4%)	24 (38.1%)	100 (36.4%)
	13–16	5 (4.8%)	6 (5.6%)	0 (0.0%)	11 (4.0%)
	>17	19 (18.1%)	19 (17.8%)	12 (19.0%)	50 (18.2%)
	Median	9	9	8	9
Sex	Male	19 (18.1%) **	21 (19.6%) *	21 (33.3%) **	61 (22.2%)
Previous treatment experience					
Ever used head louse treatment		85 (81.0%) **	98 (91.6%) **	49 (77.8%) **	232 (84.4%)
Previous treatment successful		23 (27.1%) §	**46 (46.9%) §**	14 (28.6%)	83 (35.8%)
Hair characteristics					
Length	Above ears	18 (17.1%)	26 (24.3%)	23 (36.5%)	67 (24.4%)
	Below shoulders	56 (53.3%) **	50 (46.7%)	24 (38.1%) **	130 (47.3%)
Thickness	Fine	39 (37.1%) **	43 (40.2%)	34 (54.0%) **	116 (42.2%)
	Thick	66 (62.9%)	64 (59.8%)	29 (46.0%)	159 (57.8%)
Curl	Straight	71 (67.6%)	73 (68.2%)	48 (76.2%)	192 (69.8%)
	Wavy/curly	34 (32.4%)	34 (31.8%)	15 (23.8%)	83 (30.2%)

Levels of statistical variation between groups: Figures in **bold type** show the group exhibiting a statistical disparity indicating possible randomisation anomalies. * Difference significant at p < 0.1; ** Difference significant at p < 0.05; § Difference significant at p < 0.01

### Outcomes

Post-treatment examinations at day 14 showed that there were 20/107 successful treatments and 3 cases of reinfestation after cure (an overall success rate of 21.5%) using phenothrin lotion and 18/105 successes and 3 cases of reinfestation for phenothrin mousse (giving 20.0% overall success). This made mousse users 1.07 times more likely to have lice after completion of the treatment (95% confidence interval (CI) 0.63 to 1.81; odds ratio (OR) 1.10, 95% CI 0.56 to 2.13). In the case of wet-combing with conditioner there were 12/63 (19.1%) successful treatments and no cases of reinfestation. Participants treated with combing were, therefore, 1.13 times more likely to have lice (95% CI 0.61 to 2.11; OR 1.16, 95% CI 0.53 to 2.54) than if they had been treated with phenothrin lotion. People treated with wet-combing were also 1.05 times more likely to have lice than those participants receiving phenothrin mousse (95% CI 0.56 to 1.99; OR 1.06, 95% CI 0.48 to 2.34).

In both insecticide treated groups the majority of lice at post-treatment assessments were juveniles, of which 712 first were stage nymphs that could only have originated from eggs not killed by insecticide. However, it was not possible to properly analyse the full effect of ovicidal failure due to participants being withdrawn early in the study on grounds of lack of efficacy. Nevertheless from the data available it was possible to determine that failure to kill louse eggs was a major contributing factor in the low rate of outcome success.

Intention to treat analysis found no statistical difference (p < 0.05) between the lotion and mousse. Similarly, no statistical difference was found between wet-combing and either of the insecticides. Success in curing the infestation was also not significantly associated with gender or hair type, thickness, or length, after adjustment for any randomisation anomalies. However, success rates were significantly (p < 0.01) higher in people who had previously used a head louse treatment successfully, by an estimated factor of 1.88 (95% CI 1.13 to 3.11). Success also significantly (p < 0.01) increased with age.

Analysis based on the per-protocol population, without taking into account the failure of randomisation, showed no significant difference in success rates between the two phenothrin groups (lotion 17.8%, mousse 13.7%), which showed equivalence to within 20% (mousse-lotion difference, -4.1%, 95% CI -6.1% to 14.4%).

### Adverse events

We found several clearly defined treatment-related adverse events in people treated using the phenothrin products: 12 adverse events in 11 people using lotion (9 scalp irritation, 3 irritation of the respiratory system); 10 adverse events in six people treated with mousse (5 scalp irritation, 3 dry skin, 1 bullous reaction, 1 paraesthesia of the scalp). There were no similarly defined adverse events for the wet-combing group but five carers reported children expressing signs of stress while being combed, one person reported discomfort during combing, and backache or arm/shoulder aches for three carers were also reported, but not formalised as reported adverse events. All events were considered mild and resolved rapidly except for two cases. The case of paraesthesia, which was classified as moderate, persisted for two days after treatment, and one case of dry skin persisted for some time after treatment but may have been an exacerbation of a pre-existing problem. Stinging of the hands and paraesthesia-like reactions were also reported by some of the carers while applying the phenothrin-based products. Paraesthesia has been reported from use of other pyrethroid preparations ^8– 10^ and would likely be exacerbated by the presence of alcohol in the product.

### Tests for resistance

Lice from different participants showed marked differences when tested for sensitivity to phenothrin ( Table 2). In all cases the lice were taken from people who had experienced treatment failure during the study so it was not surprising that the majority had insects that were resistant to the insecticide. However, one person had apparently also been reinfested with sensitive lice from a contact, as shown by the mixed sensitivity of the insects. All lice from another participant, treated using wet-combing, were phenothrin susceptible. Output data from the LDP Line analyses of the observations of head lice, in comparison with susceptible laboratory reared body lice, showed a resistance ratio (RR) of 54.74 when exposed to the insecticide. From the log-probit analyses the estimated time required to kill 50% of the insects (LT _50_) was 502 minutes based on a mortality curve with a slope of 1.0096 ± 0.1324 (chi-squared 19.9681, p = 0.0005). In contrast the estimated LT _50_ for the body lice was 24.74 minutes (slope = 5.1932 ± 0.5086, chi-squared = 11.6217, p = 0.0404). Log-probit analysis also suggested that the insecticide sensitive head lice (LT _50_, 44 minutes; LT _95_, 95 minutes) were approximately twice as tolerant of phenothrin as laboratory reared lice, although the number of insects involved was too small to provide a clear distinction.

**Table 2.** Sensitivity of head lice from study participants to *d*-phenothrin *in vitro*.

Participant	Treatment		Number of lice		Time for insecticide effect	
	In study	*In vitro*	Total	Killed	Knockdown	Death
18	Mousse	Phenothrin	7	[2] *	-	[540 mins] *
		Control	5	0 **	-	-
181	Lotion	Phenothrin	6	2	15 mins	45 mins
		Control	4	0 **	-	-
271	Lotion	Phenothrin	11	0 **	-	-
		Control	10	0 **	-	-
273	Mousse	Phenothrin	37	0 **	-	-
		Control	34	0 **	-	-
278	Wet-comb	Phenothrin	24	24	30 mins (6)	100 mins (23), 130 mins (1)
		Control	23	0 **	-	-
Body lice		Phenothrin	60	60	From 5 mins	15 mins (6), 20 mins (18), 25 mins (33), 30 mins (45), 45 mins (55), 50 mins (58), 70 mins (60)
		Control	60	0 **	-	-

* The time of death of these lice was sufficiently delayed that it may have been due to dehydration. ** Lice survived for longer than 540 minutes when provided with a blood meal.

Individual demographic data collected from participants and lice found by detection combing post-treatment Ethnicity: E = European, C = Caribbean, A = South Asian. Length of hair: CC = close cropped, AE = above ears, ES = ears to shoulders, BS = below shoulders. Thickness of hair: F = fine, T = thick. Straightness of hair: S = straight, SC = slight curl, C = curly. Stages of lice found: #1 = first stage nymph, #2 = second stage nymph, #3 = third stage nymph, M = male adult louse, F = female adult louse. N/A = combing not applicable.

## Discussion

This was a pragmatic study designed to investigate the three treatments under conditions that mimicked normal use by parents and carers. The outcomes demonstrated that all three products were less than adequate to eliminate head louse infestation when used according to instructions.

**Figure 2.**
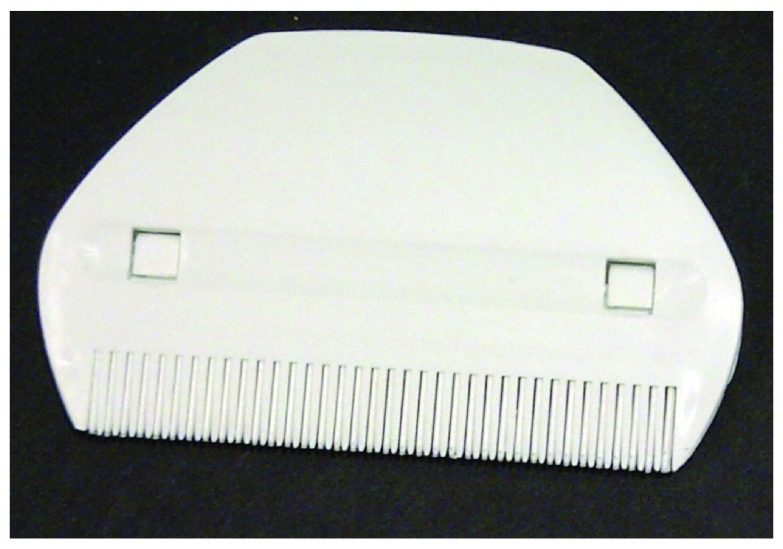
The two-part comb supplied in the Bug-Buster pack.

In the case of the phenothrin mousse, a major limitation of the product in use was that the foam was too dry and insufficiently thermo-labile. It did not break down to a fluid that could be readily spread through the hair. This problem probably arose because too little alcohol was included in the formulation so the foam generated from water and emulsifying wax did not break physically after application. Consequently, many of the parent carers probably failed to achieve an adequate or even coverage of the hair and scalp when applying it. In contrast, phenothrin lotion was too fluid, like other alcohol based products. This meant that in use it was easy to apply too little product because a small volume of the fluid made the hair look wet and, by implication, thoroughly coated. Also, this formulation did not contain any of the terpenes that had been shown to contribute much of the ovicidal activity shown by other alcoholic lotions ^11^. At that time the “Bug Buster” pack contained a two-part comb that was initially described as “unique, safe and well researched” and, “reliable even though its use may be time consuming” ^12^. Although when the product was shown to be relatively ineffective by independent investigators ^6^ they were criticised by the pack suppliers for using a product that had been superseded by the time of publication ^13^. Our investigators found it relatively easy to find lice on heads that parents using the “Bug Buster” comb believed were louse free. Therefore, it can be concluded that the two-part comb ( Figure 2) was not as effective as originally claimed and was probably not as easy to use as either the plastic detection comb we used or its replacement, which was similar to our detection comb.

For this study the sponsor engaged a general nursing agency to supply staff to perform the majority of study functions in place of trained investigators as a cost saving measure. We believe this was not only a false economy in terms of data quality but may also have resulted in a breakdown of Good Clinical Practice. We could not determine whether some of the nurses failed to understand their responsibilities as investigators, or simply could not follow instructions, but some of the apparent failures of randomisation could be attributed to poor practice. For example, people in the wet-comb group were significantly more likely to have short hair (p < 0.05) or fine hair (p < 0.05) than those in the mousse-treated group. These anomalies were attributed to a failure of randomisation at the point of enrolment by some nurses engaged in recruitment. Early in the study we drew this possibility the attention of the sponsor, as well as to the management of the nursing agency, who assured us that correct procedures were being followed. Nevertheless, a failure of randomisation was identified at analysis because we suspected a general reluctance on the part of carers and children to participate in the combing group. Boys with shorter hair were apparently seen as an easier option for wet-combing so it is possible the numbered envelopes were opened before they were specifically allocated to individuals in households were several members were participating. Therefore we adjusted for this anomaly using stratified chi-squared analyses, although ultimately it made little difference to the overall outcome analysis.

We detected another, less easily identified, anomaly in that people who had a previous experience of a successful treatment with an insecticide lotion were more likely to be recruited to the lotion group (lotion v mousse p < 0.01; lotion v wet combing p < 0.05) ( Table 1), although there was no evidence this was due to a failure of randomisation at the point of allocation.

Another deviation arose because most participants that were found to have lice at post-treatment assessments on day 4 or later were not withdrawn and provided with rescue treatment by the agency nurses but allowed to remain in the study, in some cases, until assessed by us on day 14. As far as we were able to determine, this did not affect the outcomes, and was partly associated with the logistical difficulties of transporting large volumes of documentation between offices over a short period of time, but again suggests a lack of understanding of the requirements of the study.

In the study area the local policy had been to use malathion for head louse infestation during the previous few years, so we did not anticipate significant resistance to pyrethroids. However, the small sample of lice we collected during the later stages of the study demonstrated that resistance was present and probably existed over much of the study area. The level of treatment failure could not be fully explained by resistance because five years later we observed a 75% success rate using a phenothrin aqueous emulsion in a study area that overlapped with this geographically ^14^. Rather the problem may have rested with the preparations used because another study using the alcoholic phenothrin lotion, conducted in a different part of the country, obtained a similarly poor result with only 2/15 (13.3%) cures ^15^.

This study was conducted because the Medicines Control Agency (MCA), now the Medicines and Healthcare Products Regulatory Agency, did not consider clinical investigations of the mousse in India representative of conditions likely to be encountered in Britain, so the manufacturers were required to conduct a UK-based study for confirmation of efficacy. Surprisingly however, before we could complete the analysis of this study, the MCA issued a Marketing Authorisation for the phenothrin mousse. As a result the product was launched without further assessment by the MCA. Not surprisingly, given the poor effectiveness observed in this clinical investigation, there was widespread anecdotal reporting of treatment failure by consumers after using the product. Nevertheless it remained in the UK market until 2009.

There are lessons that can be learned from this experience. The first is that bioassay tests conducted in a laboratory, whether using laboratory reared insects or even wild collected ones, can only be indicative of efficacy for a formulation ^1, 11^ and it is unknown for a treatment to perform poorly *in vitro* yet be effective *in vivo*. Of course, laboratory reared lice and other ectoparasites are usually poorly representative of the physiological characteristics of those found on their natural hosts, especially with regard to characteristics such as resistance. Even *ex vivo* screens, using insects recently collected from the wild, may be only partially representative and several replicate tests should be performed using insects from geographically separated locations to ensure that the outcome is not obtained either by chance or due to some happenstance of physiological difference in the insects from that location. Attempting to draw any kind of conclusion about efficacy from only two or three lice or a single replicate test is fraught with risk, although this appears to have been a common practice in some investigations ^16– 21^.

The second lesson, one that was recognised by the Medicines Control Agency when they initially insisted that the phenothrin mousse should be clinically tested in the UK, is that a clinical investigation of a pediculicide in a country where treatments for head lice are not routinely used is not likely to be representative of the possible outcomes in the territory where the product is destined to be marketed. Such studies may be indicative of possible outcomes but basing strong claims about how a product will work in a developed country, where lice are regularly exposed to a range of chemical entities, on the results of studies conducted in a developing country is just as flawed as relying on *in vitro* data. However, in most developed countries there are products that appear to have been evaluated only in trials in developing countries ^2, 22– 25^.

The third, and to us, the most significant point is that the efficacy data for pediculicides must be a high priority for regulatory authorities before granting a Marketing Authorisation (MA). This is significant because the products are used on children and must be safe, clinically effective, and also cost effective. In this instance the product continued to be marketed after the final data were available, despite clearly showing its lack of effectiveness. This raises a question of how many other products could be on the market without evidence of efficacy. No doubt makers of such products rely on consumer feedback and complaints as a guideline as to whether their products are both acceptable and effective. However, in practice, most manufacturers receive relatively few complaints about efficacy and few pediculicides have been subjected to the kind of post marketing surveillance applied to some other medications.

Even in the rather more litigious circumstances prevailing in the United States of America, there have been few who have gone as far as legal action to press claims of inefficacy. In overall terms it was found that the legal complaint process was hindered by the regulatory process, as argued by the defence attorneys acting for various drug companies in one class action stating “.. *The claims (of the Plaintiffs) stand in direct conflict with the Food Drug and Cosmetic Act, moreover, because the “defendants’ medications cannot be sold for the treatment of head lice and labeled to say that the medications are not effective when simultaneously federal law and regulations require the labeling to say that the products are effective.*” ^26^ ( http://www.law360.com/articles/36260/drug-makers-fight-class-action-over-lice-treatment) thereby turning the onus for verification of efficacy back to the competent authority, in this case the federal Food and Drug Administration (FDA), although in another plaint the Texas Supreme Court gave a *per curiam* ruling “... *that the FDCA contains no such “complex and interrelated federal scheme of law, remedy, and administration” that would divest the state courts of jurisdiction..*” ^27^ ( http://www.supreme.courts.state.tx.us/historical/2005/feb/031052.htm) suggesting that those courts could, if they so chose, declare products ineffective and presumably thereby place the FDA in an difficult position with respect to its approval of certain preparations ^27^. Therefore, only products for which adequate clinical studies have been conducted, and then those data placed in the public domain with appropriate periodic review to ensure resistance has not affected efficacy, can be considered effective. Simply relying on a competent authority MA is not adequate justification for continuing to sell a product when there are doubts about its effectiveness, as we highlighted in presenting the results from one of our recent investigations ^28^. Just because products or active substances may have been effective when first introduced ^29^ does not mean that they remain so, as indicated by more recent clinical investigations using some so-called “standard of care” products as comparators ^30, 31^, and both industry and regulators should be responsive to changes in circumstance.

## Data availability

*F1000Research*: Dataset 1. Individual demographic data collected from participants and lice found by detection combing post-treatment, 10.5256/f1000research.2026.d31728 ^32^

## Participant consent

Written and witnessed informed consent was obtained from the participants and parents or guardians of children under 18 years of age.

## Supplementary material

Supplementary files: https://f1000researchdata.s3.amazonaws.com/supplementary/2026/0086c068-7ac2-42a1-8135-946436252380.zip
