# Supplementary material for: Comparison of phenothrin mousse, phenothrin lotion, and wet-combing for treatment of head louse infestation in the UK: a pragmatic randomised, controlled, assessor blind trial
Source: F1000Res. 2014 Jul 10;3:158. [Version 1] doi: 10.12688/f1000research.2026.1 (PMC4168840; doi:10.12688/f1000research.2026.1)
Supplement: Peer review history

# Peer review: Referee response for version 1

**DOI:** 10.5256/f1000research.2229.r5970

**Published:** 2014-08-28

**Authors:** Maria Inés Picollo

**Affiliations:** 1 Centro de Investigaciones de Plagas e Insecticisas, Consejo Nacional de Investigaciones Científicas y Técnicas (CONICET), Buenos Aires, Argentina

This is an interesting clinical study to assess the comparative effectiveness of three head louse treatments when are used by the public. For this purpose, the treatments selected were 0.5% d-phenothrin mousse, 0.2% d-phenothrin lotion, and wet-combing with conditioner. The commercial products were applied according to manufacturer's instructions (30 minutes for 0,5% phenothrin mousse and 2 hours for 0,2% phenothrin lotion), and wet-combing according to the  Bug-Busting Community Hygiene Concern, London (fine toothed plastic comb with conditioner, repeated at 3–4 day intervals for 2 weeks).

I consider that the manuscript is within the scope of the journal, the abstract is according to the objectives and results of the manuscript: The methodology, results and discussion are carefully written and adequate to the objectives of the study.

One of the relevant aspects of the work is that reinforces the need for the effectiveness of commercial products must be warranted by the health authorities, due to the importance of pediculosis in school-aged children both in developed and developing countries.

An unexpected and surprising result is the low effectiveness of the three methods evaluated. Although pyrethroid resistance and inadequate formulation of the active ingredients surely contribute to the low effectiveness of the phenothrin-based formulations (20% and 21,5% success for mousse and lotion respectively), the low effectiveness of wet-combing (19,15 success) has not been previously reported. This result is worrying considering that the use of a fine comb for removing lice and nits was proposed as a relevant tool both in the diagnosis of infestations and as part of an integrated control strategy of head lice.

I agree with the authors in their first conclusion about the limitations of laboratory tests: ¨ *bioassay tests conducted in a laboratory, whether using laboratory reared insects or even wild collected ones, can only be indicative of efficacy for a formulation and it is unknown for a treatment to perform poorly in vitro yet be effective in vivo. ….. even ex vivo screens, using insects recently collected from the wild, may be only partially representative and several replicate tests should be performed using insects from geographically separated locations to ensure that the outcome is not obtained either by chance or due to some happenstance of physiological difference in the insects from that location*¨. But as the authors surely know and probably should clarify in the paper, the main purpose of laboratory testing is the ¨comparative-evaluation¨ of the effectiveness of pure compounds or formulations. Thus, the parallel and simultaneous evaluation of different compounds and/or formulations made ​​in standardized conditions, is a reliable result of the comparative activity of the products evaluated.

Obviously laboratory tests are faster and cheaper than clinical trials, and represent an excellent tool for a first selection of the compound or product to be developed (a pure compound without pediculicide activity in laboratory tests, will not be effective in a clinical trial).

I have read this submission. I believe that I have an appropriate level of expertise to confirm that it is of an acceptable scientific standard.

# Peer review: Referee response for version 1

**DOI:** 10.5256/f1000research.2229.r5411

**Published:** 2014-07-21

**Authors:** Deon Canyon

**Affiliations:** 1 Office of Public Health Studies, University of Hawaii, Honolulu, HI, USA

This research was conducted well, and the article was well written. There is one grammatical error in the Outcomes section (paragraph 2) : " *In both insecticide treated groups the majority of lice at post-treatment assessments were juveniles, of which 712 first were* **[1st, 2nd...]** *stage nymphs that could only have originated from eggs not killed by insecticide.*" Also, nymphs can transfer so this conclusion is not accurate. *"However, success rates were significantly (p < 0.01) higher in people who had previously used a head louse treatment successfully, by an estimated factor of 1.88 (95% CI 1.13 to 3.11)."* So how many of the failures may have been due to inappropriately applying a treatment?The title and abstract are appropriate for the article, and provide a suitable summary. The experiment appears to have been conducted properly, with appropriate controls and data measurements, and the analysis is also adequate. The conclusions drawn from the study are both sensible and balanced, and the competing interests of the authors have been sufficiently disclosed.I'm just left wondering if any of the participant variables had an influence on the outcomes? If there was no effect, this should be stated.

I have read this submission. I believe that I have an appropriate level of expertise to confirm that it is of an acceptable scientific standard.

# Response

**Published:** 2014-07-22

**Authors:** Ian Burgess

**Affiliations:** Medical Entomology Centre, UK

Thanks for finding the typographical error in Outcomes section (paragraph 2) : " *In both insecticide treated groups the majority of lice at post-treatment assessments were juveniles, of which 712 first were* **[1st, 2nd...]** *stage nymphs that could only have originated from eggs not killed by insecticide.*" that eluded everyone else reading it. 

It should have read, " *In both insecticide treated groups the majority of lice at post-treatment assessments were juveniles, of which 712 were first***** *stage nymphs that could only have originated from eggs not killed by insecticide.*"  While I do not disagree that young nymphs can transfer, this is a less common event than transfer of third stage nymphs and adults by some considerable factor of difference.  Consequently, since relatively fewer adults were found in general it seems reasonable to assume that most, if not all, newly hatched first stage nymphs originated from eggs not killed by treatment.  Consequently, I suggest a possible change to the text to read, " *In both insecticide treated groups the majority of lice at post-treatment assessments were juveniles, of which 712 were first***** *stage nymphs the majority of which most likely originated from eggs not killed by insecticide.*", which would satisfy all possibilities.

On the second point about how many treatment failures may have been due to inappropriate or incompetent application of treatment, the answer is we simply do not know.  Firstly, common sense tells us that someone who has previously successfully negotiated the pitfalls of applying a head louse treatment is more likely to achieve a similar success on a subsequent occasion.  Secondly, although the agency staff engaged by the sponsor were not experienced at performing head louse treatments so I do wonder how good they may have been (or not as the case may be) at detecting failures in application method.  Thirdly, this is a risk you take when conducting pragmatic (real use by consumer) clinical studies.  For most medications missing one dose is usually not the end of the world and does not usually affect the ultimate outcome.  However, for a single dose application head louse study not applying the product properly is usually terminal from the perspective of efficacy, irrespective of how effective the product may or may not be, and in this case they were not as effective as the sponsors believed.

So, in respect of participant variables, I do not doubt they influenced the outcome.  The difficulty remains in determining which variable had what effect.  In a wholly investigator run study, whether pragmatic or with investigator applied treatment, at least some of the variables are accountable and can be analysed accordingly.  However, in this case I don't think anyone could have done much to greatly affect the overall outcome.
